# Characteristics of hospitalizations with an influenza diagnosis, France, 2012‐2013 to 2016‐2017 influenza seasons

**DOI:** 10.1111/irv.12719

**Published:** 2020-02-05

**Authors:** Mathilde Pivette, Nathalie Nicolay, Virginie de Lauzun, Bruno Hubert

**Affiliations:** ^1^ Santé publique France Direction des régions Saint‐Maurice France

**Keywords:** epidemics, France, hospitalizations, influenza, intensive care units

## Abstract

**Background:**

Estimating the global burden of influenza hospitalizations is required to allocate resources and assess interventions that aim to prevent severe influenza. In France, the current routine influenza surveillance system does not fully measure the burden of severe influenza cases. The objective was to describe the characteristics and severity of influenza hospitalizations by age‐group and by season between 2012 and 2017.

**Methods:**

All hospitalizations with a diagnosis of influenza in metropolitan France between July 2012 and June 2017 were extracted from the French national hospital discharge database (PMSI). For each season, the total number of influenza hospitalizations, admissions to intensive care units (ICU), proportion of deaths, lengths of stay, and distribution in diagnosis‐related groups were described by age‐group.

**Results:**

Over the five seasons, 91 255 hospitalizations with a diagnosis of influenza were identified. The average influenza hospitalization rate varied from 13/100 000 in 2013‐2014 to 46/100 000 in 2016‐2017. A high rate was observed in elderlies during the 2014‐2015 and 2016‐2017 seasons, dominated by A(H3N2) virus. The youngest were impacted in 2015‐2016, dominated by B/Victoria virus. The proportion of influenza hospitalizations with ICU admission was 10%, and was higher in age‐group 40‐79 years. The proportion of deaths and length of stay increased with age.

**Conclusions:**

The description of influenza hospitalizations recorded in the PMSI give key information on the burden of severe influenza in France. Analyses of these data annually is valuable in order to document the severity of influenza hospitalizations by age‐group and according to the circulating influenza viruses.

## 1 BACKGROUND

Accurate estimation of the burden of influenza illness on hospital resources is required, both at regional and national levels, in order to allocate hospital resources and to estimate the cost‐effectiveness of specific interventions including policy decision on vaccine recommendations.

In France, severe influenza disease is routinely monitored using the number of hospitalizations following a visit in emergency departments with an influenza diagnosis.^1^ This surveillance is completed with the monitoring of clinical and virological characteristics of confirmed influenza cases admitted to intensive care units (ICU).^2^ While these surveillance systems allow a description of the dynamic of influenza epidemics, they do not provide a proper estimation of the burden of influenza epidemics on hospital activities.

Hospital discharge databases are a valuable source of information to estimate the burden of hospitalized seasonal influenza cases.^3^, ^4^, ^5^, ^6^, ^7^, ^8^, ^9^ In France, a recent evaluation of the completeness of the ICU surveillance system for influenza cases, using capture‐recapture methods, estimated the exhaustivity of the French national hospital discharge database (PMSI) to 73%.^10^ Using the PMSI database in order to estimate the burden of severe influenza illness in hospital is consequently of relevance.

The objective of this study was to assess the burden of hospitalizations with a diagnosis of influenza in France. In this aim, we described the characteristics and the severity of hospitalizations with a diagnosis of influenza by age‐group and by season over five consecutive influenza seasons from 2012‐2013 to 2016‐2017.

## 2 METHODS

### 2.1 Data sources

Data were extracted from the French national hospital discharge database (PMSI), which contains all records of hospitalizations in all public and private hospitals in France. Diagnoses are coded using the International Classification of Diseases, 10th revision (ICD‐10). We extracted all hospital stays that occurred between 1 July 2012 to 30 June 2017 in metropolitan France with at least one ICD‐10 code related to influenza (J09, J10, J11) as a principal, related or associated diagnosis. The information collected for each hospitalization was as follows: the unique patient identification number, the admission hospital identifier, the patient age, the diagnosis‐related group (DRG), the week of admission at hospital, the length of stay, whether or not an admission to intensive care unit (ICU) occurred, and the issue at hospital discharge.

### 2.2 Analysis

The analysis covers five influenza seasons from 2012‐2013 to 2016‐2017. An influenza season was defined as the period between week 45 and week 15 of the following year. Hospitalizations outside the influenza period were not included in the analysis.

In case of several stays in a same season, only one stay was kept in the final database preferably the stay with ICU admission otherwise the stay with a death record, or the stay with the most severe DRG, and the first stay if previous criteria were not discriminant.

In the database, all hospitalizations are classified in the DRG system according to diagnoses, medical procedures, and demographic characteristics of the patients.^11^ DRGs were used as a proxy of the reason of hospitalization. We defined five DRG classes: moderate influenza, severe influenza, respiratory distress, other respiratory diseases, and other DRGs.

Population size estimates were obtained from the French National Institute of Statistics and Economic Studies (2014) for the calculation of rates. Hospitalizations exceeding a 60‐days duration were excluded from the analysis of the length of stay.

For each of the five influenza seasons, the total number of hospitalizations and admissions to ICU, hospitalization rates, lethality, length of stay, and DRG distribution were described by age‐groups. We analyzed age‐groups of 20 years.

In order to describe the intensity of the epidemics within age‐groups, we classified the weekly hospitalization rate of the five influenza seasons (115 weeks) into four classes within each age‐group (<median, between median and 90th percentile, between percentiles 90th and 95th, >95th percentile).

Analyses were performed with Microsoft Excel© and R 3.4.2.

### 2.3 Definition of the virus dominance

Virological data are not available in the hospital discharge database. In order to characterize the burden of influenza hospitalizations by dominant circulating virus each season, we used virological data from primary care settings. Data were obtained from the national reference laboratory for influenza viruses. A virus type or subtype was defined as a dominant virus if (a) it accounted for 70% or more of all isolates during the season or (b) if it accounted between 40% and 70% of all isolates and the second most common virus accounted for less than 30%. Two types or subtypes were considered as co‐dominant if the main virus accounted from 40% to 70% of all isolates and the second most common accounted for 30% or more of all isolates.^12^

The dominant virus was B/Yamagata in 2012‐2013 (51%), B/Victoria in 2015‐2016 (70%), A(H3N2) in 2014‐2015 (52%), and 2016‐2017 (98%). In 2013‐2014, both subtypes A(H1N1) (50%) and A(H3N2) (42%) were co‐dominant.

Data extraction and exploitation has been authorized by the National Commission on Informatics and Liberty (CNIL n°2017‐305).

## 3 RESULTS

### 3.1 Characteristics of influenza hospitalizations by season

**Figure 1:**
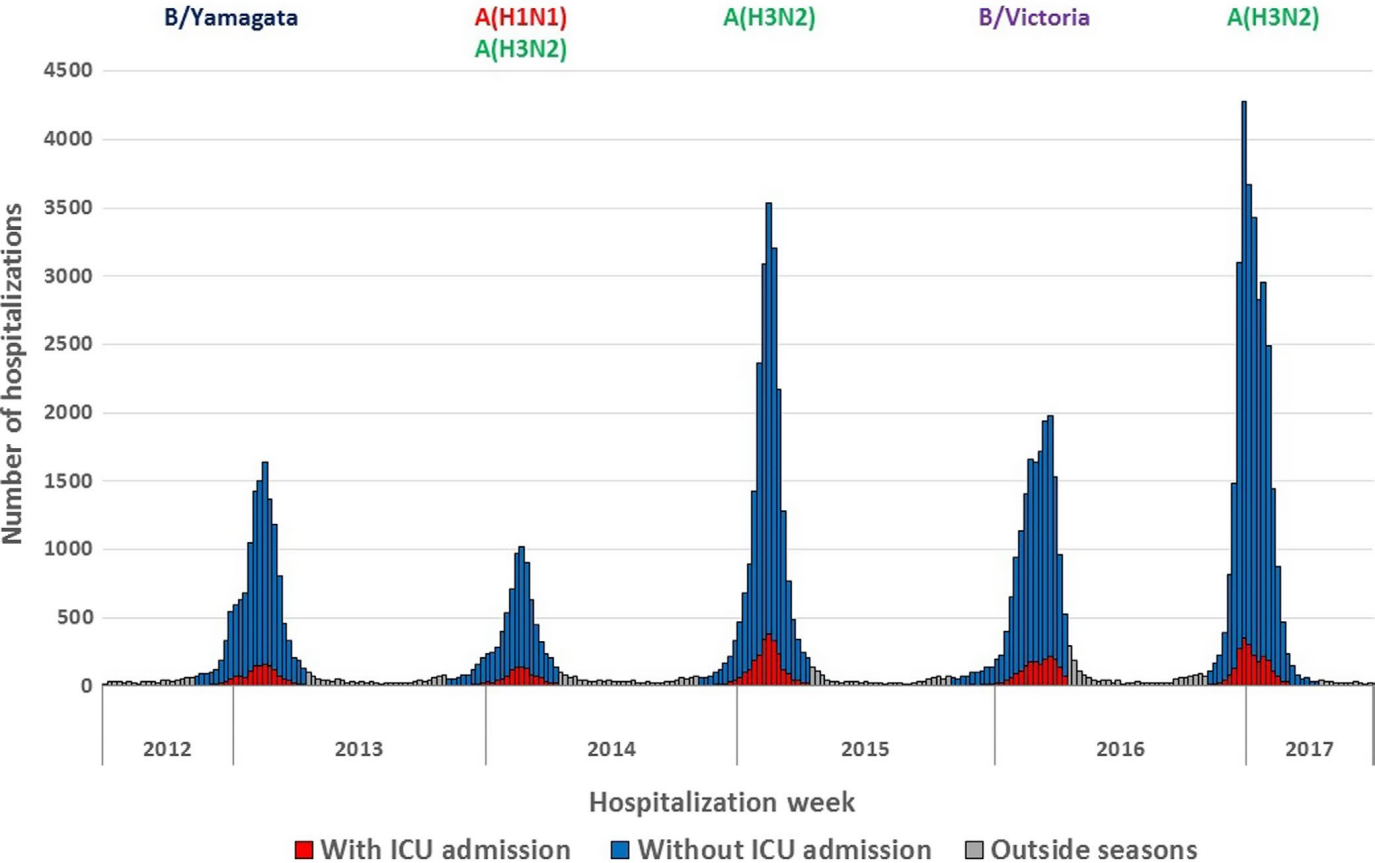
Weekly number of hospitalizations with an influenza diagnosis, 2012‐2017, metropolitan France

Between July 2012 and June 2017, 97 464 hospitalizations with an influenza diagnosis have been recorded in metropolitan France, of which 91 255 (94%) occurred during the five influenza seasons. The rate of influenza hospitalizations varied between seasons from 13/100 000 in 2013‐2014 to 46/100 000 in 2016‐2017 (Table 1, Figure 1).

**Table 1:** Description of influenza hospitalizations per season, 2012‐2017, metropolitan France

	2012‐2013	2013‐2014	2014‐2015	2015‐2016	2016‐2017	Total
Dominant influenza viruses^a^	B/Yamagata	A(H1N1) and A(H3N2)	A (H3N2)	B/Victoria	A (H3N2)	
Epidemic duration^b^ (in wk)	13	5	9	11	10	
Number of influenza hospitalizations	13 745	8 101	22 285	17 759	29 365	91 255
Hospitalization rates (/100 000)	21.5	12.7	34.8	27.7	45.9	28.5^c^
Age‐group						
<5 y	26%	27%	18%	29%	10%	20%
5‐19 y	12%	8%	6%	13%	4%	8%
20‐39 y	12%	14%	9%	14%	6%	10%
40‐59 y	17%	17%	13%	12%	8%	12%
60‐79 y	19%	21%	25%	20%	27%	23%
≥80 y	14%	13%	29%	12%	45%	27%
Number of hospitalizations with ICU admission	1 399	1 099	2 465	1 897	2 307	9 167
Proportion of hospitalizations with ICU admission	10.2%	13.6%	11.1%	10.7%	7.9%	10.0%
Hospitalization rates with ICU admission (/1 000 000)	21.8	17.2	38.5	29.6	36	28.6^c^
Number of deaths	499	338	1 220	588	1 861	4 506
Proportion of deaths	3.6%	4.2%	5.5%	3.3%	6.3%	4.9%
Number of days of hospitalization	95 087	60 709	186 014	119 483	264 059	725 352

a Source CNR of influenzae viruses.

b Source Santé publique France.

c Average rate per season.

Seasonal epidemics dominated by A(H3N2) subtype (2014‐2015 and 2016‐2017) were associated with the highest number of hospitalizations and epidemic peaks (Table 1, Figure 1).

An admission to ICU was recorded for 10% of influenza hospitalizations (n = 9167). The proportion of hospitalizations with an admission to ICU was lower when the hospitalization rate was the highest (8% in 2016‐2017) and higher when the hospitalization rate was the lowest (14% in 2013‐2014) (Table 1, Figure 1).

Overall, the proportion of deaths among hospitalizations was 4.9%. It was higher during seasons dominated by A(H3N2) viruses and lower during seasons dominated by B viruses (Table 1).

### 3.2 Distribution of hospitalizations by age

The age distribution of cases varied highly by season, both for all hospitalizations and for hospitalizations with ICU admission (Table 1, Figure S1).

Overall, the highest influenza hospitalization rates were observed in age‐group ≥80 years with an average of 134 hospitalizations per 100 000 inhabitants per season, followed by age‐groups 60‐79 years (36/100 000) and <20 years (32/100 000). Within the age‐group <20 years, the hospitalization rate was particularly high in the <5 years (93/100 000). Hospitalization rates were lower in age‐group 20‐59 years every season.

**Figure 2:**
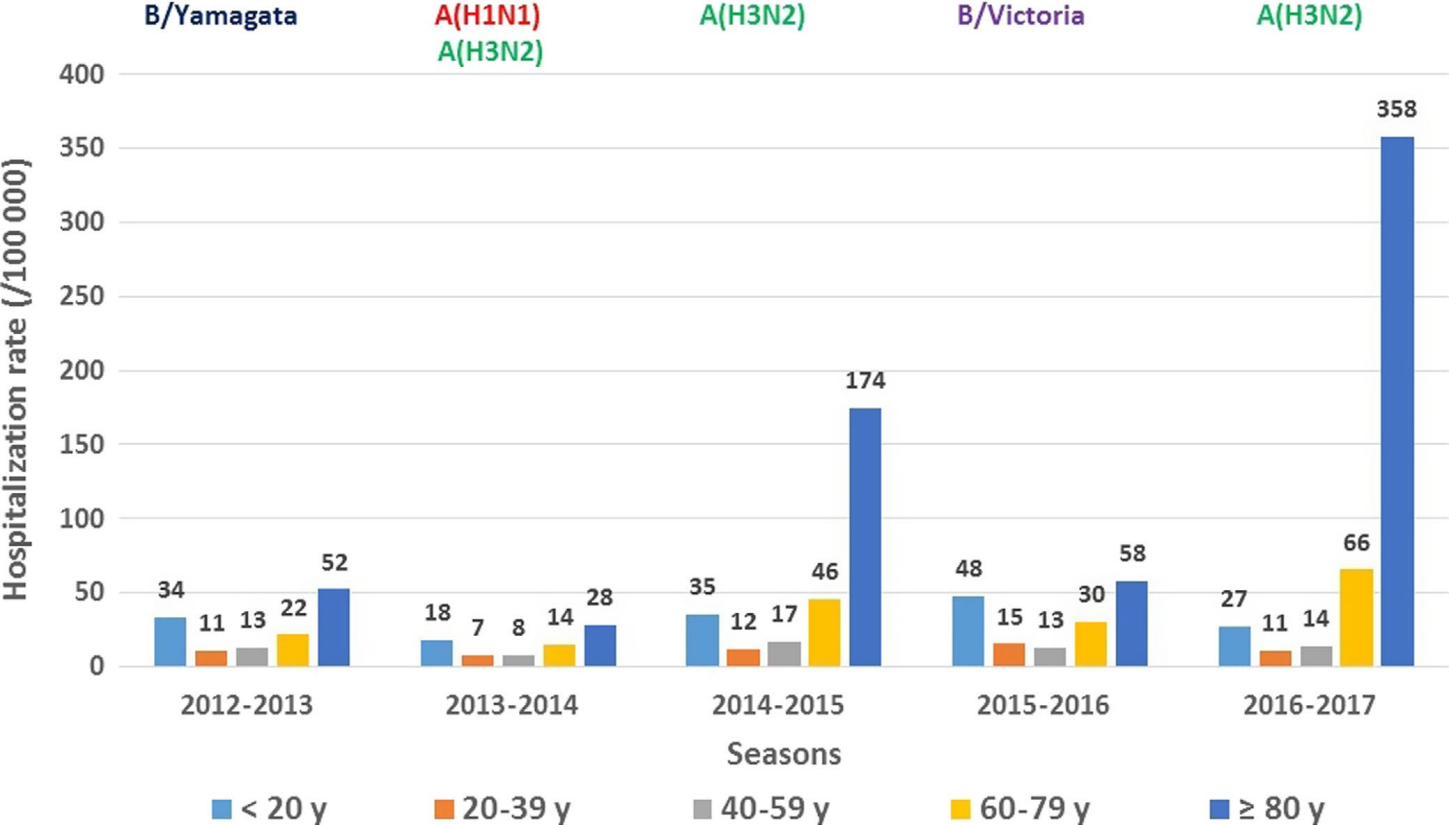
Hospitalization rate (/100 000) of influenza per age‐group and per season, 2012‐2017, metropolitan France

In age‐group ≥80 years, the highest hospitalization rates were observed during both seasons dominated by A(H3N2) viruses. In age‐group <20 years, the highest hospitalization rate was observed in 2015‐2016 dominated by B/Victoria viruses (Figure 2).

The proportion of influenza hospitalizations with ICU admission was higher in age‐group 40‐79 years (19%) compared to other age‐groups, particularly ≥80 years (6%). Few variations in the proportion of ICU admissions were observed within each age‐group across the seasons. In all age‐groups, this proportion was slightly higher in 2013‐2014 and lower in 2016‐2017 (Table 2).

**Table 2:** Proportion of hospitalizations with a stay in ICU among all influenza hospitalizations, per age‐group, and per season, metropolitan France, 2012‐2017

	2012‐2013			2013‐2014			2014‐2015			2015‐2016			2016‐2017			Total		
Dominant viruses	*B/Yamagata*			*A(H1N1)/A(H3N2)*			*A(H3N2)*			*B/Victoria*			*A(H3N2)*					
	I	H	I/H (%)	I	H	I/H (%)	I	H	I/H (%)	I	H	I/H (%)	I	H	I/H (%)	I	H	I/H (%)
<20 y	177	5260	3	91	2791	3	201	5466	4	245	7450	3	139	4229	3	853	25196	3
20‐39 y	124	1697	7	110	1148	10	137	1904	7	162	2403	7	87	1688	5	620	8840	7
40‐59 y	453	2283	20	345	1400	25	631	2984	21	521	2166	24	329	2	14	2279	11 172	20
60‐79 y	510	2569	20	463	1728	27	1048	5471	19	775	3598	22	1155	7832	15	3951	21 198	19
≥80 y	135	1936	7	90	1034	9	448	6460	7	194	2142	9	597	13 277	4	1464	24 849	6
Total	1399	13 745	10	1099	8101	14	2465	22 285	11	1897	17 759	11	2307	29 365	8	9167	91 255	10

Abbreviations: H, Number of hospitalizations; I, Number of hospitalizations with ICU admission.

**Figure 3:**
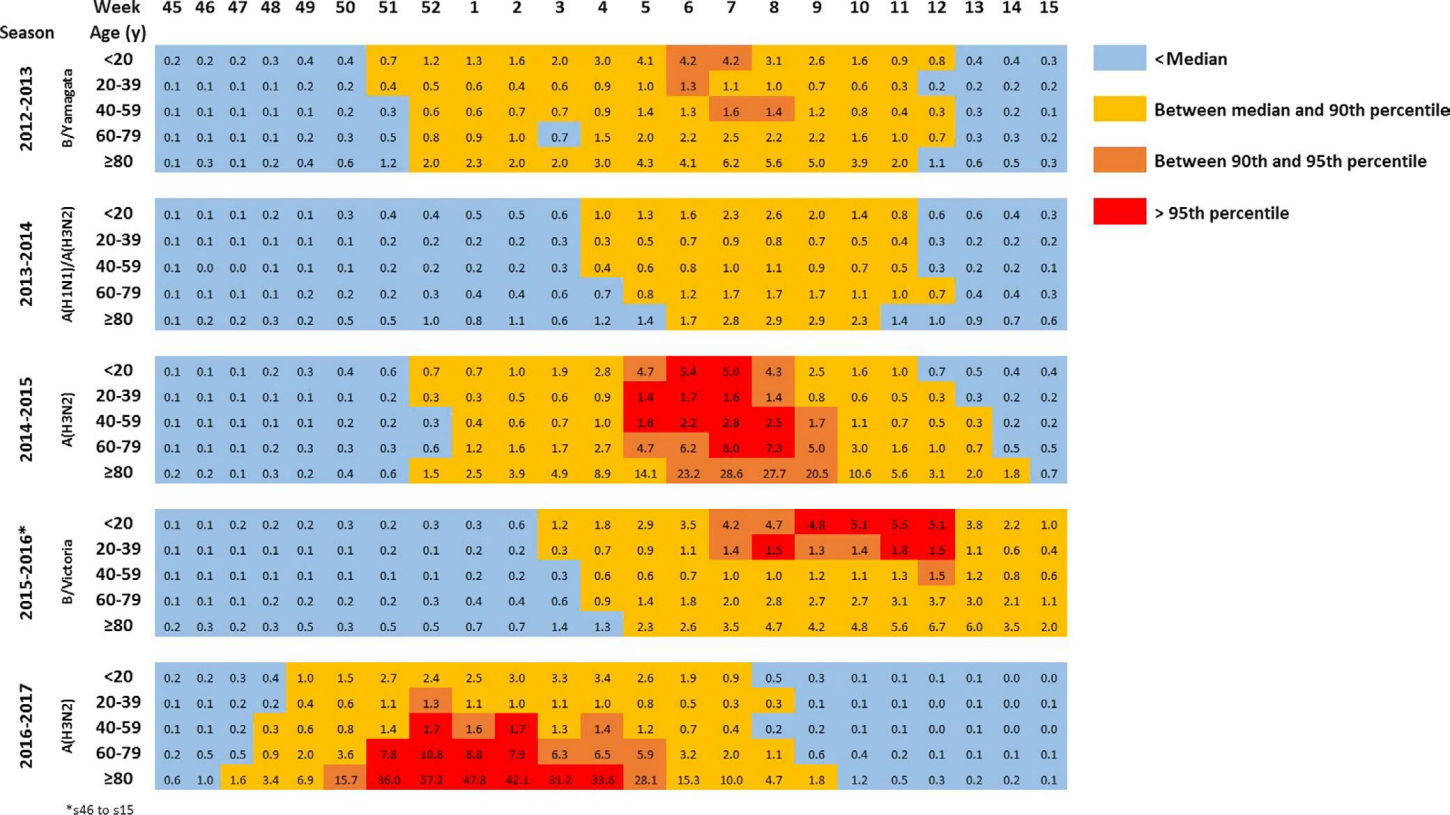
Weekly rate of influenza hospitalizations (/100 000) by age‐group and by season, and classification within each age‐group (<median, between median and 90th percentile, between 90th and 95th percentile, >95th percentile), 2012‐2017, metropolitan France

The intensity classification of influenza hospitalization rates within each age‐group illustrates the high impact of A(H3N2) dominant season in the elderlies in 2016‐2017, and of B/Victoria dominant season in the youngest in 2015‐2016. The epidemics started first in the youngest during the two seasons dominated by B viruses (Figure 3).

### 3.3 Length of hospital stay

The median length of stay was 5 days (mean = 8 days) considering all hospitalizations, respectively 15 days (mean = 18.3 days) for hospitalizations with ICU admission and 4 days (mean = 6.9 days) without (Table S1). The median length of stay increased with age‐group from 2 days in the <20 years to 9 days in the ≥80 years and was stable within each age‐group across seasons.

**Figure 4:**
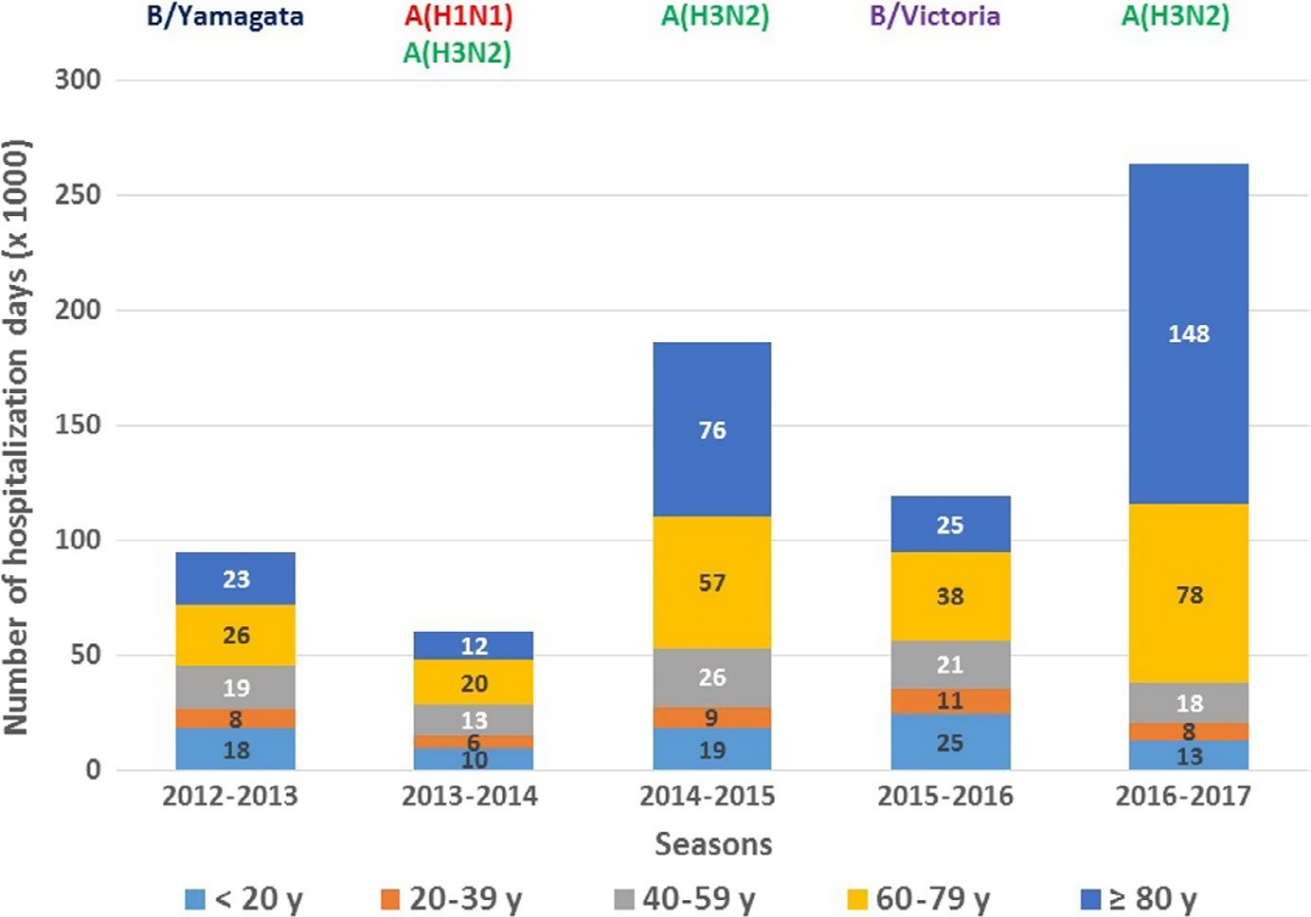
Total number of days of influenza hospitalizations by age‐group and by season, 2012‐2017, metropolitan France

The seasons with the highest numbers of hospitalization days were the two A(H3N2) dominant seasons. Over the five seasons, the ≥80 years totalized 39% of the hospitalization days, followed by the 60‐79 years (30%). The part of elderly (≥60 years) was particularly high in 2016‐2017, as they totalized 86% of the hospitalization days (Figure 4).

### 3.4 Distribution of hospital stays by diagnosis‐related group (DRG)

Among the 91 255 hospitalizations, 60% were classified in an influenza DRG, 14% in a respiratory distress or other respiratory diseases DRG and 26% in other DRGs.

The proportion of hospitalizations with a DRG of severe influenza increased with age, from 4% of the hospitalizations in age‐group <20 years to 48% in age‐group ≥80 years. Hospitalizations with a respiratory distress DRG were more frequently reported in age‐group 40‐79 years (6%) (Figure S2).

The DRGs included in the category “other DRGs” were different according to the age‐group. In the youngest, the most frequent “other DRGs” were related to neurology, with “seizure/epilepsy” accounting for 25% in the <20 years. In age‐group 20‐39 years, “other DRGs” were mostly related to pregnancy (52%). In other age‐groups, the most frequent “other DRGs” were related to cardio‐vascular conditions (18% in the 40‐59 years, 24% in the 60‐79 years, and 34% in the ≥80 years).

Hospitalizations with a DRG of respiratory distress were mostly associated with an admission in ICU (61%), as compared to the other DRG groups (1% for moderate influenza, 14% for severe influenza, 16% for other respiratory diseases, and 10% for other DRGs).

### 3.5 Lethality

Considering all hospitalizations with a diagnosis of influenza, the proportion of deaths increased with age, from 0.5% in age‐group <20 years to 10% in age‐group ≥80 years. The proportion of deaths for hospitalizations with ICU admission was 22% and increased with age, from 11% in the <20 years to 32% in the ≥80 years. Among all deaths, 54% concerned patients aged ≥80 years (Table 3).

**Table 3:** Proportion of deaths by age‐group among all influenza hospitalizations and influenza hospitalizations with ICU admission, 2012‐2017, metropolitan France

Age‐groups	All hospitalizations			Hospitalizations with ICU admission			Proportion of deaths with ICU admission (%)
	Number of deaths	Number of hospitalizations	Proportion of deaths (%)	Number of deaths	Number of hospitalizations	Proportion of deaths (%)	
<20 y	121	25 196	<1	94	853	11	78
20‐39 y	83	8840	1	70	620	11	84
40‐59 y	441	11 172	4	371	2279	16	84
60‐79 y	1445	21 198	7	1014	3951	26	70
≥80 y	2416	24 849	10	465	1464	32	19
Total	4506	91 255	5	2014	9167	22	45

Among patients younger than 80 years, most deaths occurred during hospitalizations with ICU admission (74%). In those aged above 80 years, deaths were mostly reported outside ICU (81%) (Table 3).

The proportion of deaths was stable within each age‐group across seasons (Table S2).

The median delay between hospitalization admission and death was 5 days (Interquartile range, IQR = 2‐11).

## 4 DISCUSSION

This study described the characteristics and severity of hospitalizations recorded with an influenza ICD‐10 code in the French national hospital database across five influenza seasons. Our results showed that influenza hospitalization rates by age‐group each season were highly dependent on the circulating virus. Besides, the criteria of severity (ICU admission, lethality, and length of stay) were strongly dependent on the age of the influenza cases and were quite stable within each age‐group across seasons.

### 4.1 First attempt to estimate of the influenza burden on hospitalization in France

Restricting influenza hospitalizations to hospitalizations with a diagnosis of influenza, we estimated that influenza epidemics caused 28.5 hospitalizations per 100 000 inhabitants per season between 2012 and 2017. This rate was in the lower range of figures recently published by other countries with different case definitions and data sources. Based on the analysis of national hospital discharge database, the hospitalization rate was estimated at 49/100 000 in England between 1997 and 2009,^13^ 48/100 000 in Norway between 2008 and 2017,^9^ and 33/100 000 in Canada between 2003 and 2009.^6^ In Spain, based on data from active surveillance of confirmed severe influenza cases, the hospitalization rate was 7.8/100 000 between 2010 and 2016.^5^ Despite the use of various methodologies which does not allow strict comparisons, those data confirm that influenza has a strong impact on the use of hospital resources.

### 4.2 The burden of influenza hospitalizations according to age

Previous studies highlighted the high impact of the very elderly on mobilization of hospital resources for example in England in age‐group ≥75 years 13 or in Norway in age‐group ≥80 years.^9^ In our report, a marked shift in influenza hospital burden was found in ≥80 years. Indeed, being aged 80 years and older was the paramount factor associated with the highest hospitalization rate, proportion of death, number of hospitalization days, the longest length of stay, and the highest proportion of a severe influenza diagnosis‐related group (DRG) during the study period. The impact was even more important during A(H3N2) circulating seasons in 2014/2015 and 2016/2017.^14^ Those results confirm the importance of differentiating the elderlies in several age‐groups while analyzing influenza surveillance data 15.

Noteworthy, the proportions of ICU admission in the ≥80 years each season were much lower than the proportions observed in the 60‐79 years. Also, most of deaths in the very elderly occurred outside ICU (81%). Consequently, the current seasonal routine monitoring of influenza cases admitted to ICU is less likely to capture the severity of influenza in the very elderly and underestimates the impact of seasonal influenza in this age‐group. Hence, additional data sources should be identified to monitor the impact of influenza in the very elderly in the real‐time surveillance systems.

We observed stepwise increases with age in the proportion of hospitalizations with severe influenza DRG, in the proportion of death and in the length of stay, which reflects the increased severity of influenza with age.

A quarter of the hospitalizations belonged to a DRG not related to “influenza” or “respiratory diseases”, categorized as “other DRGs”. The composition of this category greatly varied by age‐group. In young people aged 20‐39 years, a significant proportion was DRG related to pregnancy issues. In the ≥40 years, a significant proportion were cardio‐vascular conditions. We also observed the risk of influenza responsible for neurological symptoms (seizure) in the youngest. These results highlight the potentially large spectrum of influenza‐related health consequences.

### 4.3 Influenza hospitalization burden and circulating virus strain

The burden of influenza illness varied substantially according to the dominant circulating influenza subtype (Influenza A) or lineage (Influenza B) each season. Virus dominance was defined depending on the proportion of virus that was circulating each season at community level.^12^ As previously reported, the impact of specific circulating subtypes on the influenza hospitalization rates was variable depending on age^5^, ^9^, ^13^, ^16^ and previous exposition to influenza viruses. We also observed that the proportions of ICU admission may vary depending on availability of hospital resources, which depends on the total number of influenza cases during the epidemic period.

We were unable to describe the specific burden of A(H1N1) as the study period did not cover the 2009 pandemic season, and during the 2013‐2014 season, this serotype was co‐dominant with an A(H3N2) virus. However, in that particular season, the burden in the elderly was low, in line with previous findings.^9^, ^16^ This observation was attributed to repeated exposures to the virus over a 40‐year period afterward 1918^17^ resulting in a strong and long‐lasting immunity in the ≥60 years.

Compared with A(H1N1) subtype, A(H3N2) subtype usually affects all age‐groups.^18^ During the A(H3N2) dominating seasons in 2014‐2015 and 2016‐2017, our results showed that elderly were highly affected, as observed in other countries.^19^, ^20^ In 2014‐2015, the circulation of a drifted A(H3N2) strain genetically different from the northern hemisphere vaccine component was responsible for a reduced vaccine effectiveness in the population targeted by the influenza vaccine recommendations.^12^ In 2016‐2017, the global burden of influenza was even higher compared with 2014‐2015. Genetic data from virological surveillance showed that the circulating A(H3N2) viruses had undergone considerable genetic diversification responsible for high incidence and a suboptimal vaccine effectiveness in those targeted by the vaccine recommendations.^21^

Influenza B seasons accounted for a significant burden in the <20 years. Little is known about the epidemiology of B viruses distinct lineages (Victoria and Yamagata) which diverged approximately 40 years ago and have since then co‐circulated on a global scale. Victoria lineage viruses are commonly identified in school aged children.^22^ Our report showed that in 2015‐2016, the incidence in the <20 years was the highest reported over the five years study period.

The analysis of hospitalization rates stratified by age‐group illustrates the highly age‐specific impact of influenza by subtype of circulating virus. An analysis of the overall impact of influenza, without age stratification, would have masked important age‐related differences depending on the dominating influenza virus. Previous studies have shown the importance of age‐stratified analyses to assess intensity and severity of influenza.^18^ For example, in the United States, a method using intensity thresholds for multiple influenza indicators (outpatient visits, hospitalizations, and deaths) was developed to classify the severity of influenza epidemics by age‐group.^23^

### 4.4 Contribution of our study to the routine surveillance system for influenza in France

In France, real‐time surveillance of severe influenza disease is routinely monitored using the number of hospitalizations following a visit in emergency departments with an influenza diagnosis and the number of confirmed influenza cases admitted to ICU.

Our results suggest that routine surveillance of influenza does not allow a complete assessment of the burden of influenza epidemics. Hospitalizations after admission to emergency departments totalized only 24% of the influenza hospitalizations captured in the PMSI in 2016‐2017. The monitoring of influenza cases admitted to ICU does not reflect the real burden of severe disease in the very elderly who are less admitted to ICU and who mostly died outside ICU. In that sense, our analysis is of interest to describe the burden of influenza hospitalizations each season and should be repeated annually.

The real‐time analysis of PMSI database is not possible as the delay of data availability is too long (at least 6 months). It would therefore be interesting to consider the application of multiplier method to correct and to extrapolate real‐time surveillance data in order to estimate the influenza disease burden each season.^24^

### 4.5 Main limitations

Our study presents the usual limitations associated with the use of medico‐administrative databases with coding rules based on economic and non‐epidemiological criteria.

Our definition of influenza hospitalization was thought to be very specific as we assumed that the attribution of an ICD‐10 influenza code relies on a viral confirmation test as per guidelines recommendations. A previous study also showed that the exhaustivity of the hospital database for the surveillance of influenza cases admitted in ICU with a confirmed influenza diagnosis was about 73%.^10^

While patients with influenza may present with various clinical pictures or clinical complications at a later stage, it may happen that no virological confirmation is performed^25^, which underestimate the burden of influenza hospitalizations. However, no data on influenza testing practice in hospital settings are available in France to confirm it. More information on testing behaviors are necessary in order to interpret the variations in influenza hospitalization rates over the study period.

Our results reflect the burden of cases with a diagnosis of influenza requiring hospitalization and consequently does not give a comprehensive picture of the global influenza burden which should also include influenza patients hospitalized without confirmation diagnosis, patients cared in emergency departments and community healthcare settings as well as the influenza attributable deaths. Overall, the burden of hospitalizations with a diagnosis of influenza is only part of the entire burden.

## 5 CONCLUSION

The analysis of hospitalizations with an influenza diagnosis using the national hospital database provides important information on the burden of influenza epidemics. Analyses of these data annually would be valuable in order to document the severity of influenza hospitalizations by age‐group and according to the circulating influenza viruses. To get a more complete picture of the hospitalization burden related to influenza, further analyses need to be performed on acute respiratory infection hospitalizations without influenza diagnosis.
