# Supplementary material for: Characteristics of hospitalizations with an influenza diagnosis, France, 2012‐2013 to 2016‐2017 influenza seasons
Source: Influenza Other Respir Viruses. 2020 Feb 5;14(3):340–8. doi: 10.1111/irv.12719 (PMC7182605; doi:10.1111/irv.12719)
Supplement: Supplementary file 1 [file IRV-14-340-s001.docx]

**Supplementary material**

Figure S1: Total number of influenza hospitalizations (A) and influenza hospitalizations with ICU admission (B) per age and per season, 2012-2017, metropolitan France

Figure S2: Distribution of diagnosis related groups (DRG) for influenza hospitalizations by age-group, 2012-2017, metropolitan France

Table S1: Median and mean length of stay in days of influenza hospitalizations by age-group over the study period, 2012-2017, metropolitan France

|  | With ICU admission | | Without ICU admission | | All hospitalizations | |
| --- | --- | --- | --- | --- | --- | --- |
| Age-groups | median [IQR] | Mean (sd) | median  [IQR] | Mean (sd) | median  [IQR] | Mean (sd) |
| < 20 years | 9 [5-18] | 13.7 (12.4) | 2 [1-3] | 3 (3.8) | 2 [1-4] | 3.4 (4.7) |
| 20 - 39 years | 11 [6-23] | 15.9 (13.3) | 3 [1-5] | 3.9 (4.3) | 3 [1-5] | 4.7 (6.2) |
| 40 - 59 years | 16 [9-28] | 19.7 (14.1) | 4 [2-8] | 6.1 (6.7) | 5 [2 -10] | 8.7 (10.1) |
| 60 - 79 years | 16 [9-27] | 19.1 (13.4) | 6 [4-11] | 8.7 (8) | 7 [4-13] | 10.5 (10) |
| ≥ 80 years | 15 [8-24] | 17.9 (13) | 9 [5-14] | 11.1 (8.5) | 9 [6-15] | 11.5 (9) |
| **Total** | **15 [8-26]** | **18.3 (13.5)** | **4 [2-9]** | **6.9 (7.5)** | **5 [2-10]** | **8 (8.9)** |

*IQR: Interquartile range
Sd: standart deviation*

Table S2: Proportion of deaths by age-group and by season for influenza hospitalizations, 2012-2017, metropolitan France

|  | 2012-2013 | | | 2013-2014 | | | 2014-2015 | | | 2015-2016 | | | 2016-2017 | | | Total | | |
| --- | --- | --- | --- | --- | --- | --- | --- | --- | --- | --- | --- | --- | --- | --- | --- | --- | --- | --- |
| Dominant viruses | B-Yamagata | | | A(H1N1)/A(H3N2) | | | A(H3N2) | | | B-Victoria | | | A(H3N2) | | |  | | |
|  | D | H | D/H | D | H | D/H | D | H | D/H | D | H | D/H | D | H | D/H | D | H | D/H |
| *< 20 years* | 26 | 5260 | **<1%** | 16 | 2791 | **1%** | 30 | 5466 | **1%** | 36 | 7450 | **<1%** | 13 | 4229 | **<1%** | 121 | 25196 | **<1%** |
| *20 - 39 years* | 20 | 1697 | **1%** | 16 | 1148 | **1%** | 15 | 1904 | **1%** | 17 | 2403 | **1%** | 15 | 1688 | **1%** | 83 | 8840 | **1%** |
| *40 - 59 years* | 91 | 2283 | **4%** | 62 | 1400 | **4%** | 130 | 2984 | **4%** | 94 | 2166 | **4%** | 64 | 2339 | **3%** | 441 | 11172 | **4%** |
| *60 - 79 years* | 198 | 2569 | **8%** | 137 | 1728 | **8%** | 377 | 5471 | **7%** | 271 | 3598 | **8%** | 462 | 7832 | **6%** | 1445 | 21198 | **7%** |
| *≥ 80 years* | 164 | 1936 | **8%** | 107 | 1034 | **10%** | 668 | 6460 | **10%** | 170 | 2142 | **8%** | 1307 | 13277 | **10%** | 2416 | 24849 | **10%** |
| ***Total*** | **499** | **13 745** | **4%** | **338** | **8 101** | **4%** | **1220** | **22 285** | **5%** | **588** | **17 759** | **3%** | **1861** | **29 365** | **6%** | **4506** | **91 255** | **5%** |

*D: Deaths
H: Hospitalizations*
